# Supplementary material for: Balancing selection at the human salivary agglutinin gene (DMBT1) driven by host-microbe interactions
Source: iScience. 2022 Apr 1;25(5):104189. doi: 10.1016/j.isci.2022.104189 (PMC9038570; doi:10.1016/j.isci.2022.104189)
Supplement: Dcoument S1. Figures S1 and S2 and Table S1 [file mmc1.pdf]

iScience, Volume *25*

Supplemental information

Balancing selection at the human salivary agglutinin gene (*DMBT1*) driven by host-microbe interactions

Adel F. Alharbi, Nongfei Sheng, Katie Nicol, Nicklas Strömberg, and Edward J. Hollox

Supplementary figures

Supplementary Figure 1   Haplotype network of the *DMBT1* 16kb region in the French population, Related to STAR Methods

Haplotype median joining network of GRCh38 chr10:122555466-122571966 showing balancing selection and high Tajima’s D values. The line indicates the boundary between haplotypes carrying C and A at rs11523871 and A, h indicates observed haplotypes, with size of circle representing number of observations in the sample, MV indicates internal nodes with no observed representative haplotype.

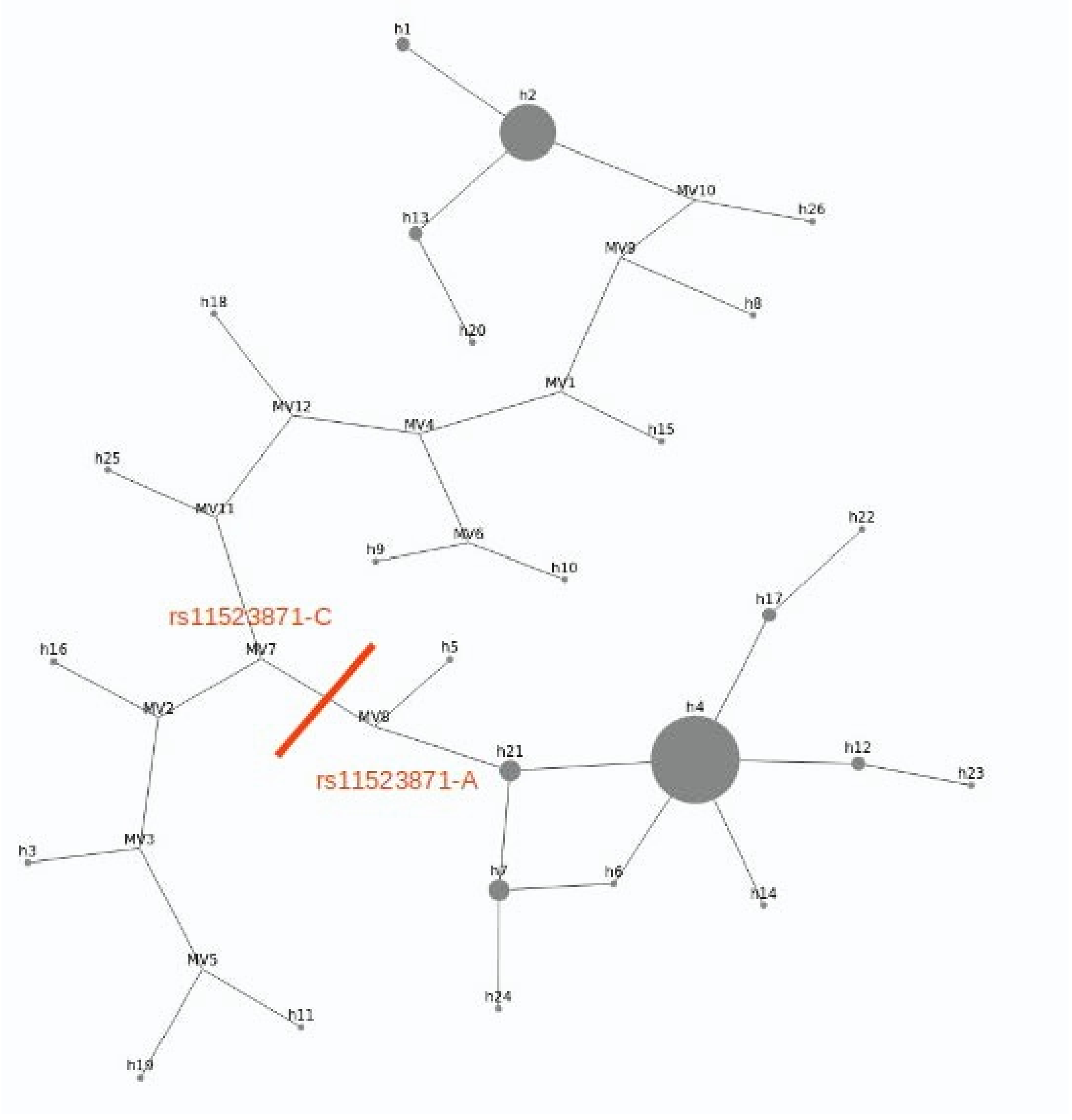

Supplementary Figure 2 Binding of *S. mutans* phenotypes and recombinant rCnm to DMBT1 and co-receptors in saliva, Related to Figure 5.

Saliva proteins blotted to a membrane after separation of parotid saliva were overlaid with wildtype (spaP A+, cnm+), single (spaP A- or cnm-) and double (spaP A-, cnm-) knock-out mutants and rCnm. The wildtype strain and Cnm+, spaP- mutant and rCnm protein showed equal binding to DMBT1, while the spaP A+, Cnm- and spaP A-, cnm- mutants showed reduced but residual activity. Both strains, mutants and rCnm bound to mono- and dimer amylase but strains/mutants and not rCnm to the acidic PRP protein bands, suggesting a plausible unspecific binding to the major saliva amylase components but potentially specific features of the acidic PRP co-receptors. The amylase and PRP components are marked by arrows.

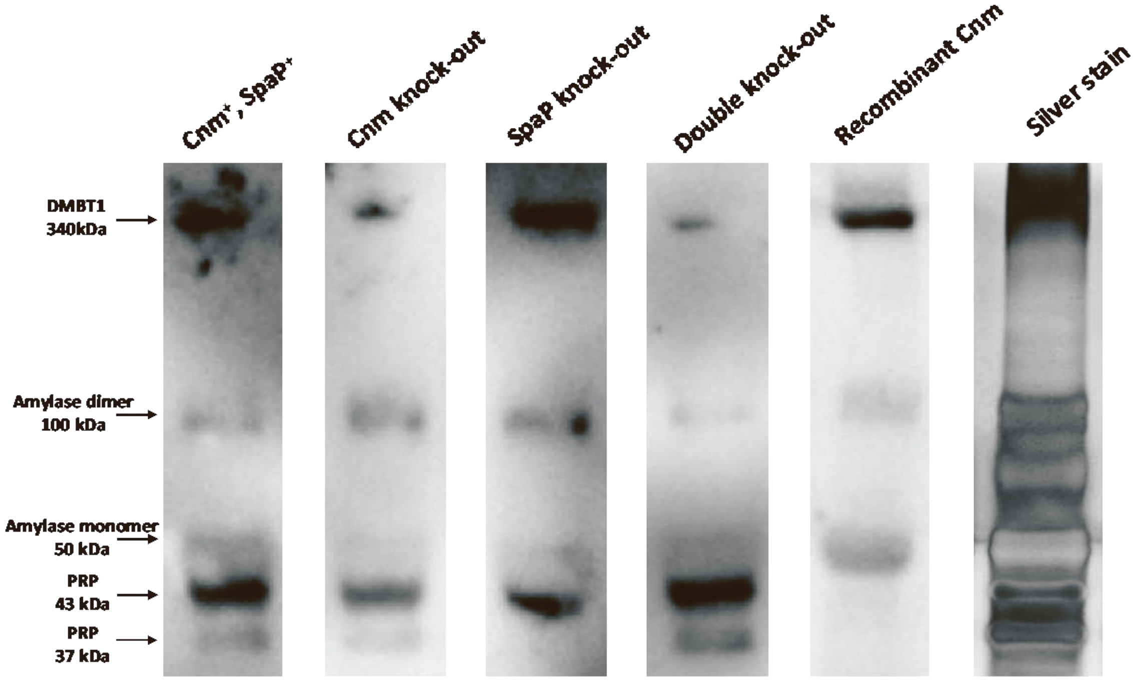

Supplementary table

Supplementary Table 1 - Normalised values for *DMBT1* Tajima's D in HGDP populations GRCh38 chr10:122555466-122571966, related to STAR Methods

Population	z_value_TajimaD
Adygei	2.76
Balochi	1.49
Bantu_NE	3.22
Bantu_S	3.75
Bedouin	3.27
Biaka	3.44
Brahui	2.00
Burushi	1.08
Cambodian	2.36
Dai	0.64
Daur	-0.27
Druze	1.73
French	3.36
French_Basque	3.63
Han	1.19
Hazara	0.75
Hezhen	-1.25
Japanese	0.95
Kalash	-0.01
Lahu	-0.07
Makrani	2.15
Mandenka	4.02
Maya	-1.41
Mbuti	1.90
Miaozu	2.13
Mongola	-0.32
Mozabite	4.21

NAN_Melanesian	-0.94
Naxi	-0.50
North_Italian	0.55
Orcadian	2.33
Oroqen	-1.15
Palestinian	3.11
Papuan	-1.78
Pathan	2.16
Pima	-1.95
Russian	2.77
San	1.60
Sardinian	3.41
She	2.45
Sindhi	0.96
Tu	1.00
Tujia	2.28
Tuscan	1.90
Uygur	1.77
Xibo	-0.41
Yakut	0.51
Yizu	0.94
Yoruba	4.29
